# Extracellular Vesicles as Nanoparticle Delivery Vectors in Cancer Therapy

**DOI:** 10.1021/acs.nanolett.6c00911

**Published:** 2026-06-26

**Authors:** Maria Sancho-Albero, Ana Martín-Pardillos, Jose L. Hueso, Victor Sebastian, Jesus Santamaria

**Affiliations:** † Instituto de Nanociencia y Materiales de Aragón (INMA), CSIC-Universidad de Zaragoza, Campus Rio Ebro, Edificio I+D, C/Poeta Mariano Esquillor, s/n, Zaragoza 50018, Spain; ‡ Department of Chemical and Environmental Engineering, University of Zaragoza, Campus Rio Ebro, C/María de Luna, 3, Zaragoza 50018, Spain; § Networking Research Center in Biomaterials, Bioengineering and Nanomedicine (CIBER-BBN), Instituto de Salud Carlos III, Madrid 28029, Spain; ∥ Instituto de Investigación Sanitaria (IIS) de Aragón, Avenida San Juan Bosco, 13, Zaragoza 50009, Spain; ⊥ Departamento de Bioquímica y Biología Molecular y Celular, Facultad de Ciencias, 16765 Universidad de Zaragoza, 50009 Zaragoza, Spain; # Facultad de Ciencias de la Salud y del Deporte, 16765 Universidad de Zaragoza; Plaza Universidad 3, Huesca 22002, Spain; ∇ Escuela Politécnica Superior, 16765 Universidad de Zaragoza, Crta. de Cuarte s/n, Huesca 22071, Spain; ○ Laboratorio de Microscopías Avanzadas, 16765 Universidad de Zaragoza, E-50018 Zaragoza, Spain

**Keywords:** Extracellular vesicles (EVs), tumor targeting, extracellular vesicle engineering, nanoparticle delivery

## Abstract

Three decades after the approval of the first cancer nanomedicine, low (<1%) tumor delivery remains the central unsolved challenge in nanoparticle (NP)-based therapy. This barrier has prompted a research shift toward biologically derived delivery systems able to reduce immune clearance while preserving tumor-homing capabilities. In particular, extracellular vesicles (EVs) seem obvious candidates on account of their intrinsic biocompatibility, cell-specific tropism, and biological functionality. In this mini-review, we critically analyze EVs as nanoparticle delivery vectors in cancer therapy. We describe current EV engineering approaches, including loading methodologies, surface modification strategies, and the development of artificial or biomimetic EVs, highlighting technical, scalability, and characterization challenges. We also summarize key *in vitro* and *in vivo* results, addressing encapsulation strategy, biodistribution, and therapeutic outcomes. From this discussion, we outline research needs that must be addressed to develop EV–NP hybrids as tools to overcome the delivery challenge in cancer.

## Graphical abstract

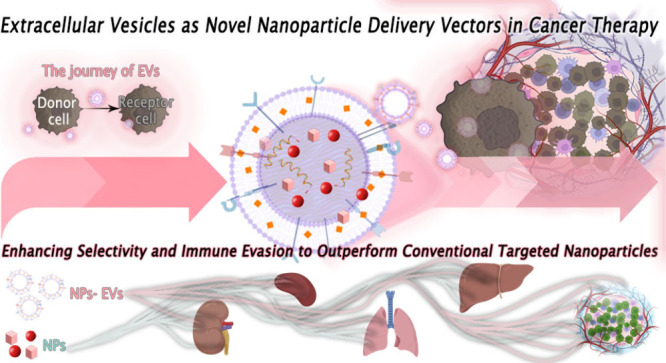

In spite of extraordinary advances in almost every branch of medical sciences, cancer continues to be a huge health concern, with 21.3 million new cases and 10.4 million cancer-related deaths in 2025,^1^ and a 64% increase predicted for 2050, reaching over 35 million cases per year.[Bibr ref2] 1995 saw approval of the first cancer nanomedicine, Doxil, a pegylated liposomal formulation containing doxorubicin, raising huge expectations. The reasoning was that, if Doxil, a passive vector, had shown enough efficiency to attain approval, surely a new generation of smart, actively targeted nanoparticles (NPs), could end cancer. Researchers worldwide responded with a myriad of NPs functionalized with a wide variety of targeting moieties (antibodies, peptides, aptamers, etc.), able to recognize cancer-associated epitopes, internalize in cells, escape endosomal degradation or exert therapeutic functions in response to external stimuli or to signals from the tumor microenvironment.

Now, three decades after Doxil and thousands of sophisticated NPs later, we know better. While many exciting nanoformulations reached clinical trials,^3a^ most failed at phases II and III. Even for those cancer nanomedicines that attained approval, the criticism was that they mainly contributed to reducing side effects, rather than improving therapeutic efficacy.^3b^

## The 1% Reality Check

The main problem of cancer nanomedicine became evident in 2016, when Wilhelm et al.^4^ published their landmark analysis of the literature, showing that less than 1% (in fact, a median value of 0.7%) of systemically administered NPs reached the tumor, ending instead in other organs, mainly the liver. This happened regardless of the nature of the particles and whether passive or active targeting was used. Four years later, an independent analysis^5^ not only confirmed the previous report but also found no significant improvement of tumor delivery efficiency, despite new NP developments in the post-2016 period. These data^4^ accelerated a revision of hypotheses in targeted nanomedicine that were already underway. To start with, passive targeting, based on the enhanced permeability and retention (EPR) effect, i.e., the concept behind the first (Doxil) and all the subsequently approved cancer nanomedicines, was challenged.^3a,3b^ However, active targeting did not fare better. Already in 2010, two separate studies demonstrated that modifying Au NPs with targeting moieties such as transferrin^6a^ or trastuzumab^6b^ did not significantly improve tumor accumulation with respect to the unmodified NPs. Among the many studies confirming this trend, that of Dai et al.^7^ was especially relevant: using NPs coated with trastuzumab or folic acid, they demonstrated not only that a small fraction of NPs reached the tumor (again, 0.7% of total dose), but that the majority of NPs reaching the tumor were trapped in the extracellular matrix (ECM) or uptaken by Tumor-Associated Macrophages (TAMs), and only 0.0014% of injected dose actually reached tumor cells.

The dismal landscape of cancer nanomedicine is in strong contrast with the success achieved by nanovectors in the wake of the COVID-19 pandemic, when decades-long research efforts in nanotechnology could be leveraged to produce with unprecedented speed new vaccines against SARS-CoV-2,^8^ at the same time sparking record-breaking investment in RNA-based therapies.^9^ However, unlike COVID-19 nano-enabled vaccines, cancer nanomedicine requires site-specific delivery, i.e., the NPs must reach a precise location in the body (the tumor), with high selectivity (to avoid devastating off-target side effects) and in sufficient amounts (to avoid development of treatment resistances). Today, it is widely recognized that the specific targeting of tumors is still an unsolved nanomedicine challenge.^10^

## The Tumor Targeting Problem

The first obstacle that systemically injected NPs have to overcome is escape from phagocytosis. A large majority of NPs (up to 95%) end up in the liver^4,11^ mostly captured by Kupffer cells (KCs), the resident macrophages in the liver sinusoids (Figure 1a). This is not surprising: the anatomy of the liver provides almost perfect conditions for NPs capture. Blood from the portal vein and hepatic artery is directed to a network of sinusoids, tiny channels of 7–15 μm diameter,^12^ of which a human liver contains around a billion.^13^ Blood flow in the sinusoids is extremely slow, with velocities of 200–800 μm/s,^14^ giving ample opportunity for NP uptake by KCs. It is important to remark that the problem of NPs capture by KCs in the liver cannot be avoided by anchoring targeting moieties on the NPs. If anything, targeting ligands, especially antibodies, are suspected to help macrophage detection, reducing circulation times.^6b,10^ Other approaches such as pegylation are effective in delaying KC uptake and increasing blood circulation time, although NPs nevertheless accumulate in the liver over time.^15^ Alternatively, “self” marker moieties have been attached to the NP surface as “do not eat me” signals. In particular with CD47, a transmembrane protein up-regulated in cells of hematopoietic lineage has provided some success in reducing macrophage uptake.^16^ Other, more drastic, but effective means to reduce macrophage uptake target the macrophages themselves, by saturating them with NPs^17^ or directly killing them.^18^

**Figure 1:**
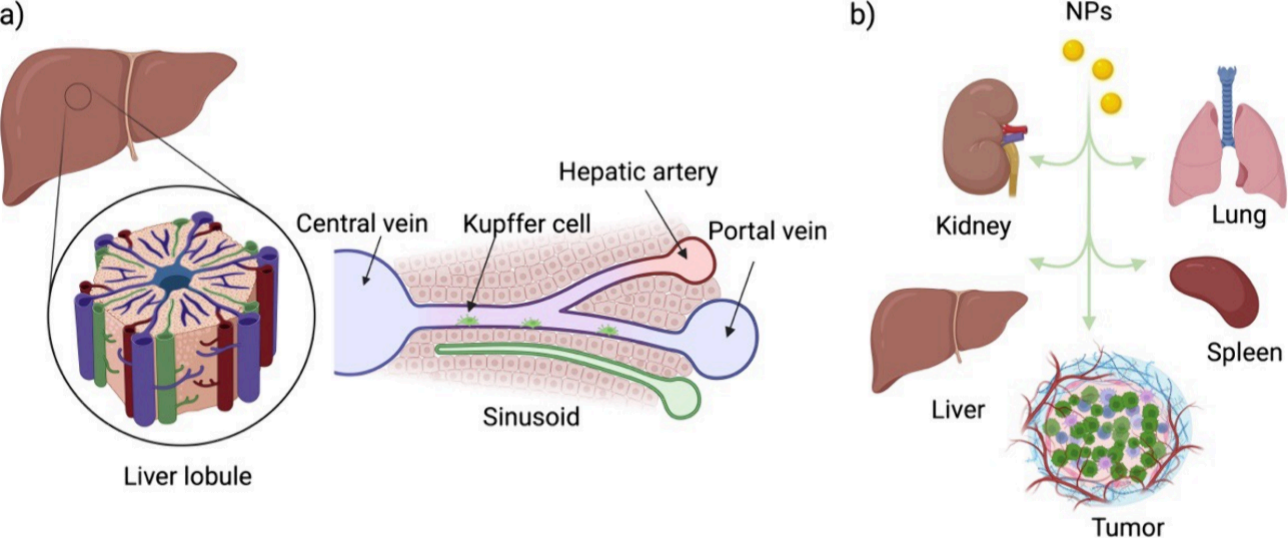
Tumor targeting problem. (a) The phagocyte challenge: most NPs (up to 99%) end up in the liver sequestered by resident macrophages (Kupffer cells). (b) The targeting challenge: many cells ad organs compete with tumor cells as final NP destination. Created with www.biorender.com.

Even if we had KC-evading NPs capable of achieving long blood circulation times, accurate targeting is needed, as many organs compete with tumors as final destinations for NPs in circulation (Figure 1b), causing off-target toxicity. It seems clear that EPR-based passive targeting cannot provide enough selectivity, and active targeting has not been able to make up for its shortcomings in spite of a wide variety of targeting moieties: antibodies, antibody fragments, aptamers, proteins, peptides, carbohydrates, and small molecules such as folic acid or biotin.^19^ Part of the problem of active targeting is that no specific receptors are exclusively displayed by tumor cells. Therefore, to increase the probability of delivery to the desired cells, NPs must be engineered with a sufficient density of targeting moieties to promote multiple NP-cell surface interactions^20^ or even with diverse targeting ligands.^21^ However, as already noted, a high density of targeting ligands is in conflict with avoiding detection by macrophages. Active targeting is also compromised by the biomolecular corona (BC) formed around NPs in biological environments, which interferes with the ability to bind to specific receptors on tumor cells.^22^ Because of these unsolved problems, no actively targeted NPs have yet been approved for clinical use in oncology.

The above summary makes it clear that avoiding uptake by resident macrophages in the liver (and to a lesser extent in the spleen) while retaining sufficient tumor-targeting capabilities is a formidable task. Faced with this challenge, researchers have started to exploit the so-called Trojan horse strategies^23^ to obtain tumor-homing NP delivery systems with a decreased immune system recognition. This mini-review specifically addresses extracellular vesicles (EVs) as perhaps the most promising of NP delivery vectors and discusses current efforts to obtain NP-EV hybrids with sufficient tumor targeting capabilities. The work is focused on NP delivery strategies, and thus, other approaches (e.g., NP-mediated RNA delivery) are not considered within its scope.

## Successful NP Delivery to Tumors

A universally accepted definition of successful delivery does not exist, and also, there are obviously no therapeutic indexes defined for NPs. However, there are benchmarks from different perspectives that are useful as references. Especially interesting are data from clinically relevant settings with patients, often using radiolabeled drugs. Thus, for instance, Areberg et al.^24^ monitored ^191^Pt-labeled Cisplatin in patients with different tumors (bladder, testicles, lung, rectal, oral) and found accumulations of 1 to 4.7 mg/g of tumor tissue for an 180 mg administration of cisplatin, meaning that the absolute percentage of drug delivered to the tumor was well below 1% of injected dose. Data from animal models, where the determination can be done with a high precision, confirm this. Dawidczyk et al.^25^ studied the tumor accumulation of LipoCure liposomal doxorubicin (an improved analogue of Doxil) in different tumor models and found accumulations ranging from 0.4% (colorectal) to 0.2% (breast) to 0.05% (pancreatic) of injected dose (ID) in the corresponding xenografts, i.e., accumulations strongly under 1%. Also, Obata et al.^26^ used radiolabeled Pt, to show that accumulation of IV-supplied free cisplatin in the tumor mass reached approximately 0.6% to 1.0% ID/g, meaning that for a typical-size tumor, the absolute percentage of the total dose reaching it would again be clearly below 1%.

In this context, given the fact that chemotherapy drugs (even when supplied as liposomes, leveraging the EPR effect) show intravenous delivery efficiencies below 1% ID, it seems reasonable to consider that delivery of therapeutic nanoparticles above 1% ID can be considered as successful, provided that the NPs involved can sustain prolonged activity, such as catalytic or hyperthermia-enabling nanoparticles, where repeated therapeutic action (many events per nanoparticle) is possible as long as the delivered nanoparticle remains active.

## EVs as a Potential Solution for a More Effective Tumor Targeting

### EVs: Definition, Biogenesis, and Classification

EVs are membrane-derived vesicles released from all kinds of cells, from humans to plants and bacteria.^27^ Although initially they were described as cellular garbage bins, currently their role as messengers between cells is widely accepted as well as the potential that EVs traffic presents to deliver therapeutics in medical applications.^27,28^

Classically, EVs have been divided in several classes: apoptotic bodies, microvesicles and exosomes according to their biogenesis pathway and size^27,28^ (Figure 2). Exosomes, also called small EVs, are vesicles that range from 30 to 150 nm in diameter and are generated by inward invagination of the late endosomal membrane to form the multivesicular body (MVB). Through their formation, exosomes can include a representative cargo of their parental cells, such as endosomal proteins, cytosolic proteins, and specific proteins and genomic compounds.^27,28a^ The enrichment on certain proteins, as tetraspanins (CD63, CD9 and CD81), membrane trafficking proteins (RAB proteins and annexins) and proteins involved in MVB formation (ALIX, TSG101 and clathrin) have been classically employed to characterize exosomes. Large EVs are divided on microvesicles and apoptotic bodies. Both are produced through the outward budding of the cell membrane, the critical difference between them is the cell state: microvesicles are secreted from completely functional cells, while apoptotic bodies are secreted from dying cells, leading to distinct contents. Sizes are also different 100–1000 nm and 500–5000 nm for microvesicles and apoptotic bodies, respectively.^27,28a^ Microvesicle cargo includes biomolecules that are translocated to the plasma membrane, rRNA (rRNA) and mRNA (mRNA).^28a^ Annexin A1 has been described as a specific marker for microvesicles budding from the plasma membrane.^29^ Finally, apoptotic bodies may contain whole cellular organelles, nuclear genomic DNA, fragmented nucleic acids and randomly enclosed cargo, trapped during the blebbing of the membrane.^28a^ While the above classification is often used, recent guidelines from the International Society for Extracellular Vesicles (ISEV)^30^ encourage to use the general denomination “Extracellular vesicle” since many separation and purification methods yield EV populations with overlapping size profiles and cannot separate efficiently EVs produced by different biogenesis pathways. In addition, the lack of universal molecular markers to distinguish among different types of EVs means that their study is often complex and experimental results obtained with EVs may reflect the combined action of more than one vesicle type.

**Figure 2:**
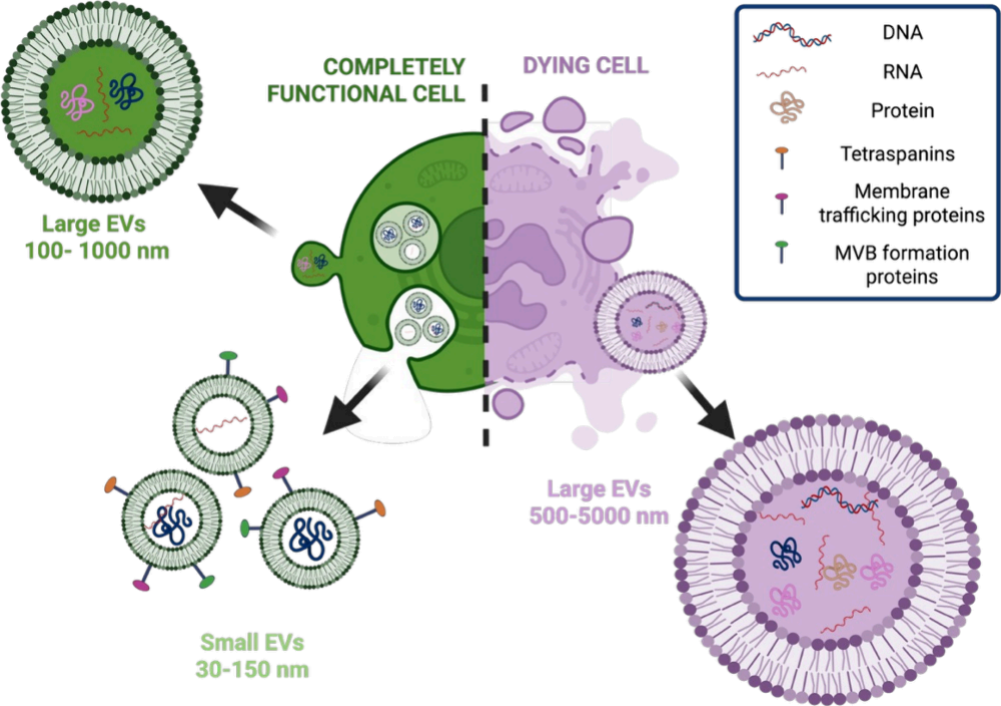
EVs are membrane-derived vesicles released from all kinds of cells. EVs are divided according to their biogenesis pathway. Small EVs (30 to 150 nm) are generated by inward invagination of the late endosomal membrane to form the multivesicular body (MVB), include a representative cargo of their parental cells and are enriched on certain proteins, as tetraspanins, membrane trafficking proteins and proteins involved in MVB formation. Large EVs are produced through the outward budding of the cell membrane and can be divided on microvesicles (range from 100 to 1000 nm) and apoptotic bodies (500–5000 nm) depending on the state of the secreting cell (fully functional/dying). Microvesicle cargo includes biomolecules such as RNA and proteins, while apoptotic bodies comprise whole cellular organelles, DNA and randomly enclosed cargo. Created with www.biorender.com.

### Potential Use of EVs as Delivery Systems—The Journey of EVs from the Donor Cell to the Receptor Cell: Migration through Barriers and Immune Evasion.

Among the many biological processes involving EVs, there is compelling evidence of their key role in cancer dissemination and metastatic growth.^31^ In doing so, EVs must succeed in overcoming a series of formidable barriers and obstacles. First, they are released from primary tumor cells and must migrate through the pericellular and then extracellular matrix (ECM)^32^ to arrive at the systemic circulation. Then they must cross endothelial barriers, evade immune system removal, reach the metastatic niche, and the targeted cells while surviving physical and biological degradation on the way. To achieve this, EVs use a variety of strategies. For instance, it has been reported that they are able to release matrix-remodeling enzymes, playing an active role in remodeling the ECM.^33^ Also, experiments *in vitro* demonstrated that EVs were able to cross a layer of brain microvascular endothelial cells by transcytosis (EVs internalization and exocytosis of newly formed EVs at the other side), especially under stroke-like conditions.^34^ In addition, they have been shown to promote degradation of endothelial cell junctions as a way to increase vascular permeability by increasing reticulum stress in human umbilical vein endothelial (HUVECs) cells^35a^ or by acting as carriers of cancer-secreted miR-105.^35b^ Finally, EVs display substantial targeting selectivity, dictated largely by their surface integrin profiles.^27^ In view of the above features and especially of the potential of EVs for selective delivery, it is not surprising that researchers have attempted to hijack EVs’ traffic to convey NPs to tumors, in spite of the significant challenges to be overcome (Figure 3).

**Figure 3:**
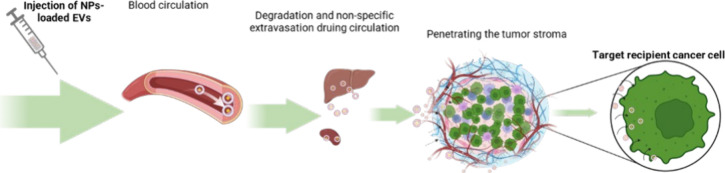
Schematic representation of challenges involved in the journey of NP-loaded EVs after systemic administration. Most of them are likely to be uptaken by phagocytes and accumulated in nontarget organs. The fraction of NP-loaded EVs that successfully reach tumoral tissue must penetrate the ECM and then home on cancer cells (and not in other cells such as fibroblasts or tumor-associated macrophages) and be internalized. Created with www.biorender.com.

Selective EV-mediated delivery can be achieved under suitable conditions. It has been demonstrated that EV origin determines to a large extent cell targeting and the transfer of EVs content *in vitro.* ^36^ However, things can be very different *in vivo*, where the complexity of the environment is orders of magnitude higher and EVs must cross biological barriers and evade phagocytosis (Figure 3). Systemically injected EVs appear to migrate to specific organs, although stochastic addressing is also supported by the literature.^37^ Several studies have identified proteins involved in tropism to particular tissues and cells, including tetraspanins, integrins, lipids, lectins, heparansulfate proteoglycans and ECM components.^27^ As examples, exosomal integrins α6β4 and α6β1 have been associated with lung metastasis, exosomal integrin αvβ5 targets the liver^38a^ and integrin-binding sialoprotein (IBSP)^38b^ and integrin αv have been related with induction of osteolysis to initiate the premetastatic niche.^39^

In any case, to reach targets located at long distances, EVs must evade the immune system, or at least reduce the likelihood of detection and removal. This is favored by the fact that EVs include in their surface specific proteins, such as integrin ß3, ICAM-1 and surface glycosphingolipids (as the CD24 ^40a^ and CD47 ^40b^), that help to protect EVs from degradation by the mononuclear phagocyte system (MPS).^41^ However, the capacity of EVs to avoid detection and removal by the MPS is being questioned.^42^ On the one hand, there is solid evidence that EVs are also subjected to phagocytosis and undergo rapid clearance from circulation.^43^ On the other, this evidence comes mainly from studies using “exogenous” EVs, i.e., works where EVs are isolated, labelled with a dye or protein and then injected in an animal model to study biodistribution. It seems reasonable that the labelling itself may induce changes in surface chemistry, charge and aggregation state of the EVs (see some problems with lipophilic dyes).^44^ There is also the question of the “bolus effect”, i.e., whether the biodistribution studies, typically involving one or more point injections of a large amount of EVs, adequately represent the real scenario with a low-intensity continuous release of EVs.^42,45^

#### Transfer of the EV Cargo to the Receptor Cell

Once EVs reach their target, their therapeutic cargo must be transferred to the cell cytoplasm. Three main mechanisms have been described: endocytosis, direct fusion, and receptor–ligand binding.^27,37,46^ Regarding endocytosis, described pathways include clathrin-dependent endocytosis, clathrin-independent pathways such as caveolin-mediated uptake, macropinocytosis, phagocytosis, and lipid-raft-mediated internalization. EV size has an effect on the internalization route.^47^ For instance, uptake of isolated exosomes and small EVs seems to occur by micropinocytosis, whereas large EVs or small-EV aggregates too large for this pathway may be internalized through alternative mechanisms.^27^ Then, a variety of effects have been described, although there is no consensus regarding the likely outcomes related to specific surface features. As examples, the protein tetherin has been reported to facilitate phagocytosis or micropinocytosis favoring uptake of large masses or aggregates of EVs.^48^ Also, specific structures at the plasma membrane of the target cell, such as filopodia that drive EVs toward uptake sites or the presence of lipid rafts, seem to contribute to EV internalization, while their disruption by cholesterol depletion reduces uptake of EVs.^27^

After endocytosis, the release of the EV cargo relies on the pH-dependent fusion between the EV and endosome membranes. A family of interferon-induced transmembrane proteins has been related with the regulation of content release by this specific mechanism.^46^ A number of EVs surface proteins have been related with their uptake, such as tetraspanins, integrins, lipids, lectins, proteoglycans, and ECM components and T cell immunoglobulin and mucin domain containing protein 4 (Tim4).^27,37^ Additionally, the presence of externalized phosphatidylserine seems determinant for EV recognition and control the transfer cargo.^49^ In this line, several studies have shown that depletion and digestion of EV surface proteins significantly reduce EVs internalization in recipient cells.^39b,50^ However, no protein alone seems to be sufficient to guarantee EV internalization and cargo release, which seems to depend instead on multiple-membrane-based interactions. Therefore, it seems reasonable to assume that membrane characteristics must be preserved as much as possible to favor both specific targeting and the ability to release content in the receiving cell.

#### Biomolecular Corona

It is now widely accepted that when artificial NPs interact with biological systems, endogenous biomolecules bind to their surfaces, leading to the formation of a new biological identity known as the BC. A few years ago, BC was reported for retroviruses^51^ (with similarities to EVs in terms of nanometric size, lipid membrane, and interaction with recipient cells^52^) and its existence is now increasingly acknowledged also for EVs.^53^ As discussed above, for EVs, the general belief was that their membranes played a central role in the recognition processes involved in cell-to-cell communication. However, the existence of a BC on top of the EV membrane poses obvious questions regarding its effect on EV-cell interactions. Indeed, some data suggest that the formation of a biomolecular around EVs could significantly influence their targeting properties^53^ and their biological effect^54^ in the recipient cells through yet largely unexplored mechanisms. Thus, the traditional view of EVs as membrane-enclosed carriers whose targeting properties are solely membrane-dependent becomes too simplistic. Instead, the rational design of next-generation EV-based therapies needs to recognize the formation of the BC and leverage its influence on processes such as immune evasion and target cell recognition.

#### Loading EVs with Nanoparticles

The incorporation of NPs into EVs while preserving as much as possible the membrane features remains a major challenge, given the large size of NPs compared to small molecules or nucleic acids and the structural complexity of EV membranes. From a methodological perspective, NP loading strategies can be broadly classified into pre-EV isolation (endogenous) and post-EV isolation (exogenous) approaches (Figure 4a), each associated with specific advantages and limitations, including scalability issues.

**Figure 4:**
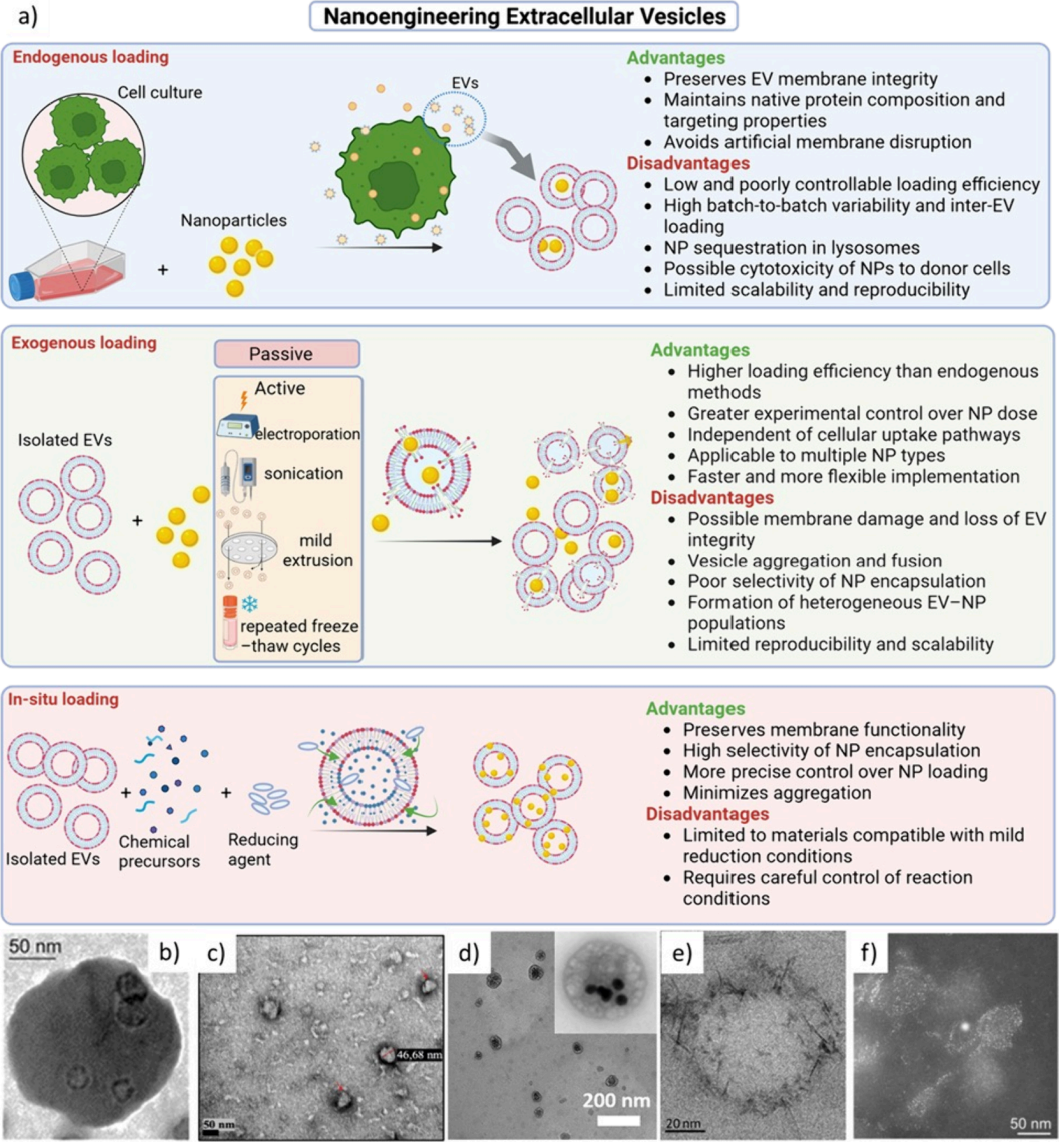
(a) Schematic overview of the main strategies to load EVs with NPs, together with their main advantages and limitations. Created with www.biorender.com. (b–f) Representative transmission electron microscopy (TEM) images of EV–NP systems obtained using different nanoengineering strategies. (b) EVs derived from B16–F10 cells after incubation with PEGylated hollow Au NPs, (endogenous loading). Adapted from ref 55 with permission. Copyright Royal Society of Chemistry (2019). (c) EVs from MSCS cells endogenously loaded with chlorophyllin-based carbon dots. Adapted from ref 56 under CC-BY-4.0. Copyright American Chemical Society (2025). (d) EVs derived from MCF-7 cells loaded with TAT-peptide-modified vanadium carbide quantum dots (V_2_C QDs) via electroporation (active exogenous loading). Adapted from ref 57. Copyright American Chemical Society (2019). (e) EVs from A549 cells engineered with Pd nanosheets via an in situ method. Adapted from ref 58. Copyright Springer Nature (2019). (f) EVs from B16–F10 cells loaded with Pt NPs using the in situ synthesis approach. Adapted from ref 59 under CC-BY-4.0. Copyright Springer Nature (2022). Note: Quantitative data on encapsulation efficiency and NP per EV or post purification yields are generally not provided in the revised works; therefore, the statements made (e.g., relative comparisons of loading efficiency) represent the authors’ opinion, rather than a quantitative analysis.

Endogenous or “natural” loading uses the EVs biogenesis pathway to load NPs into EVs, most commonly through coincubation of cells with the NPs. While this strategy has been extensively and successfully applied for the loading of molecules (e.g. nucleic acids^60^), its translation to NPs faces more difficulties. Indeed, the process yields NP-loaded EVs (Figure 4b, c), but it is typically low-efficiency and hard to control, resulting in highly variable loadings (Figure 4a). A representative example is the internalization of anti-miR-21-conjugated gold NPs into EVs.^61^ Although conceptually elegant, the loading efficiency was low and the process would be difficult to scale, highlighting the limitations of this approach. Moreover, internalized NPs often accumulate in lysosomes, where they may undergo degradation before EV secretion. Importantly, NP surface chemistry plays a critical role in this context. In our own experience, pegylation of 40 nm hollow Au NPs significantly improved their endogenous loading efficiency (Figure 4b), whereas non-pegylated NPs showed minimal incorporation.^55^ Also, NP aggregation state, surface chemistry and colloidal stability are critical parameters to consider. A variety of exogenous loading methods with the same hollow Au NPs exhibited poor loading efficiency and caused significant damage to the EV membrane. It must be noticed that these NPs have a relatively large size (ca. 40 nm) compared to small EVs (50–150 nm), meaning that a higher intensity of the method was required to achieve significant loading. In summary, the natural method can be used with a variety of NPs including carbon quantum dots,^56^ porous silicon NPs^62^ or semiconductor quantum dot^63^ (Figure 4c) and represents probably the best option for preserving membrane integrity, though at the expense of low loading efficiency, limited control, and poor scalability.

Exogenous loading methods, performed after EV isolation and purification, offer greater experimental control over NP loading, as they bypass cellular machinery and intracellular processing pathways. Exogenous approaches can be divided into *passive* and *active* techniques (Figure 4a). Passive loading relies on diffusion across the EV membrane driven by concentration gradients and is effective for small, lipophilic molecules. However, due to size constraints and membrane impermeability, this strategy is generally ineffective for NPs. An interesting exception involved the successful loading of 5 nm glucose-coated Au NPs into EVs via the GLUT-1 glucose transporter in the EVs, demonstrating that membrane transporters can occasionally be exploited for small NP internalization.^64^ In contrast, active exogenous loading strategies aim to transiently disrupt the EV membrane to facilitate cargo entry. The method must be applied with a low enough intensity to keep membrane disruption reversible and facilitate recovery of membrane properties. Commonly used methods include electroporation, low-intensity sonication,^65^ mild extrusion, and repeated freeze–thaw cycles (Figure 4a).^55,66^ Among these active loading strategies, electroporation and sonication remain the most widely used. Thus, electroporation was used to load vanadium carbide (V_2_C) dots into MCF-7 EVs^57^ and 5 nm superparamagnetic iron oxide NPs into EVs in the presence of trehalose^67^ (Figure 4d), and sonication was used to coat silica NPs with AS1411-modified macrophage exosomes^68^ and magnetic mesoporous silica NPs^69^ with tumor cell EVs membranes, although in this latter case, vortexing was additionally employed. Coextrusion of EVs and NPs has been also explored to ensure the encapsulation of a plethora of NPs, for instance, Au NPs,^70a^ porous silicon NPs,^70b^ MnO_2_ NPs^71^ and Prussian Blue (PB) NPs^72^ However, in many such cases, the actual loading efficiency is difficult to quantify accurately, especially it is often hard to ascertain whether the NPs are actually internalized in EVs, associated with their outside or embedded in a matrix of membrane fragments. More importantly, although active methods can enhance cargo incorporation, they often induce vesicle aggregation, membrane fusion, loss of membrane proteins or structural damage,^67^ raising concerns regarding functional preservation, reproducibility and scalability. Thus, exogenous approaches provide greater control over loading and are easier to scale up, but often compromise EV integrity.

A conceptually different strategy is the *in situ* synthesis of noble metal NPs inside EVs.^73^ In this case, metal ion precursors are first internalized into EVs through passive diffusion, followed by a purification step to remove noninternalized ions. Subsequently, a mild gaseous reducing agent (typically CO) is introduced at room temperature, which diffuses across the EV membrane and reduces the metal ions, triggering nucleation and growth of NPs within the EVs (Figure 4e, f). This strategy appears to preserve the fundamental properties of the exosomal membrane: protein composition, targeting capability, and biological functionality. For instance, it yielded EV–PdNP hybrids that retained preferential tropism toward their parental cells^58^ while displaying catalytic properties *in vitro* ^58^ and *in vivo.* ^74^ Notably, *in vivo* Pd-EVs (Figure 4e) accumulated more efficiently in tumor tissue than Pd-pegylated NPs, while decreasing off-target deposition in the liver.^74^ This method can be applied to any metal precursor that can be reduced by CO and was extended to platinum-based systems,^59^ generating ultrasmall (<2 nm) Pt NPs inside EVs (Figure 4f). *In vivo*, the resulting PtNP-loaded EVs exhibited antitumoral efficacy comparable to cisplatin while markedly reducing systemic toxicity. A different approach was used by Li et al.,^75^ who combined *in situ* synthesis and natural biogenesis to achieve internal synthesis of tellurium NPs. In their study, Te NPs were biosynthesized intracellularly in H22 hepatoma cells leading to intrinsic encapsulation during EV biogenesis. The resulting TeNPs-EVs provided a NIR-triggered photothermal response to activate HSP70, enhancing immunological response and improving antitumor efficacy. Taken together, *in situ* synthesis methods offer an alternative strategy that helps to maintain EVs functionality while increasing loading homogeneity compared to endogenous methods. However, their applicability remains limited to cases where precursors can diffuse through biological membranes and then be reduced in the inner space.

#### Artificially Built EVs: Membrane Cloaking and Hybrid EV–Mimetic Systems

Artificial EVs provide a completely different alternative to the above strategies, aiming for a greater reproducibility, process control, and scalability.^76^ As shown in Figure 5, a wide array of possibilities exists, from fully artificial EVs, where no natural component is employed to membrane-cloaked NPs where only natural membrane fragments are used, with EV-based hybrids occupying an intermediate space. For coatings using natural membranes, numerous works have used cell membranes (e.g., from cancer cells,^77^ mesenchymal stem cells^78^ or neutrophils^79^) to obtain a biomimetic coating on NPs. However, here we will focus only on works using EV membranes as a biological shell.

**Figure 5:**
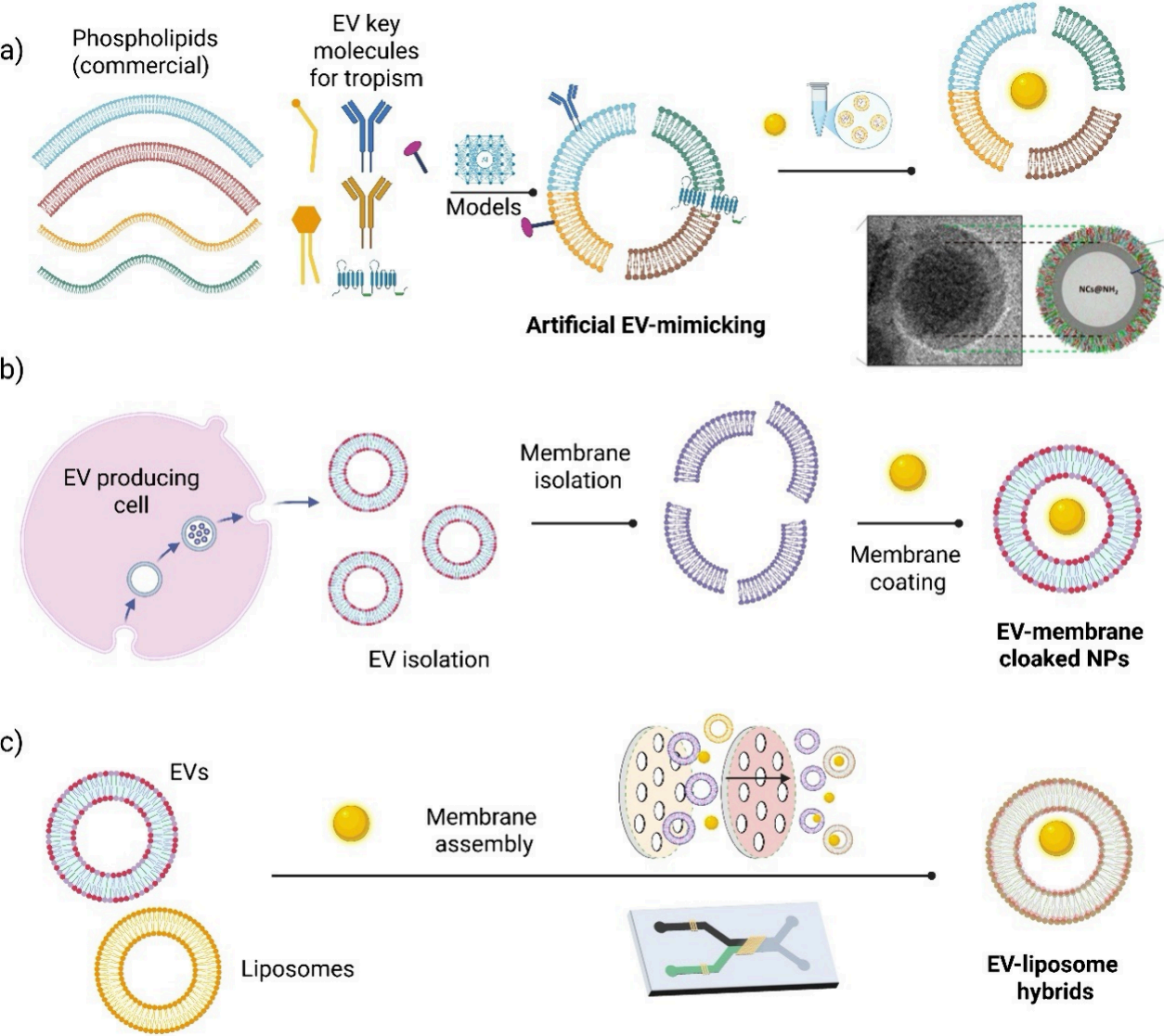
(a) Artificially built EV-mimicking coatings. Inset shows TEM images of SiO_2_ NPs encapsulated in modeled mixtures of commercial phospholipids. Adapted from ref 80 under CC-BY-4.0. Copyright American Chemical Society (2025. (b) EV membrane-cloaked NP.; (c) EV–liposome hybrid vesicles. Images created with www.biorender.com.

Fully artificial EVs (Figure 5a) are typically generated through bottom-up approaches, starting from liposome-, proteoliposome-, or lipid-coated NP-like formulations. Their composition can be rationally tuned by selecting key lipids, cholesterol, targeting ligands, fusogenic components, or purified/recombinant membrane proteins. This approach allows fine control over size, surface charge, membrane composition, and cargo loading and increases batch-to-batch reproducibility. As a recent example, organosilica nanocapsules were coated by using lipid mixtures designed from EV lipidomic data. The objective was not to reproduce the full molecular complexity of native EVs but to obtain an artificial construct that could approximate biophysical properties relevant to cellular recognition.^80^

In the second group (Figure 5b), only EVs membranes are used as coating. In this architecture, the NP is not enclosed inside an intact EV. Instead, the EV membrane is harvested and reassembled around the NP surface, in a core–shell structure. This presents a key advantage when cancer cells EVs are used as membrane donors (Figures 5b and 5c): in the process of harvesting membranes, most of their biological content, that has been related to cancer progression, can be removed.^76,81^ After collection of EVs membranes, a method to efficiently putting in contact the membrane fragments and the NPs is needed. Microfluidic coating offers the prospect of a continuous, better-controlled process compared to batch sonication, but its successful application requires that the resulting EV membrane-coated NPs are true core–shell hybrids rather than mixtures of vesicles and particles.^77a,77c^

Finally, hybrid EV-mimetic systems, incorporate biological material derived from EVs together with synthetic lipids or liposomes (Figure 5c). The rationale is to combine the inherent biological features of EVs membrane fragments with the higher formulation control, loading capacity and scalability of liposomes. Several strategies have been reported to facilitate their formation, including repeated freeze–thaw cycles, coextrusion through porous membranes, direct incubation of EVs and liposomes, sonication, incorporation of EVs or EV-derived components during thin-film hydration, and microfluidic mixing or microfluidic-assisted fusion. Among these, coextrusion and sonication can promote efficient membrane disruption and reassembly, but they may also affect the integrity or orientation of EV membrane proteins.^76,82^ Freeze–thaw cycles are experimentally simple and have been used to induce EV–liposome fusion, although they provide limited control over size distribution and lamellarity. Direct incubation is milder and easier to implement, but fusion efficiency can be low and strongly dependent on lipid composition, membrane charge and incubation conditions. Thin-film hydration enables EV-derived components to be incorporated during liposome formation but faces a difficult scalability. Finally, microfluidic approaches offer a continuous and potentially scalable alternative, with improved reproducibility,^76,82^ but faces other problems such as sample dilution, and the possibility of shear stress induced damage. Thus, the synthesis method can have a strong influence on the final architecture, membrane composition and biological performance of the hybrid construct.

In summary, artificial EVs show great potential for their scalability and translation. However, expecting that a membrane built artificially around NPs with EV membrane fragments and/or with suitable chemical molecules will reproduce the same features, as the original EV membrane does not seem realistic. Instead, a more convincing goal would be to achieve a coating that approaches some of the desirable functions attributed to EV membranes, mainly preferential targeting and a reduced immune system response. Postcoating characterization should include structural imaging, membrane protein profiling, and, more importantly, functional verification with basic experiments, e.g., *in vitro* delivery selectivity or macrophage evasion efficiency. Membrane orientation, protein density, and postcoating corona formation remain underexplored variables that can strongly influence these biological outcomes.^82,83^

## Results Obtained with NP-Loaded EVs

### *In Vitro* and *In Vivo* Studies: Selectivity According to Cell Origin

#### Unloaded EVs

Although the preferential uptake of cancer-derived EVs by their parental cells is demonstrated *in vitro* and *in vivo*,^84,85^ the limited understanding of how they are taken up by cells continues to hinder the development of more effective EV-based therapies. Studies that compare targeting efficiencies *in vitro* and *in vivo* are especially valuable because they address the intrinsic delivery selectivity for a certain type of EV and its performance in a more complex environment where delivery selectivity compounds with other factors such as extravasation or immune system capture. It must be taken into account that the very process of loading EVs with NPs may alter their behavior. However, the next two examples illustrate the difficulties of drawing clear conclusions even from empty (unloaded) EVs.

EVs from colorectal cancer (C26-derived EVs) were internalized more efficiently by C26 donor cells than by melanoma B16-BL6 recipient cells (31.1 vs 23.6%). A similar pattern was observed for B16BL6-EVs, which showed higher uptake in B16BL6 donor cells compared to C26 recipient cells (59.6% vs. 42.6%). This relatively modest preferential uptake *in vitro* did not translate *in vivo*, where both C26-EVs and B16-BL6-EVs accumulated primarily in the liver during the first 72 h, and no accumulation was detected in the tumors of C26 tumor-bearing mice.^86^ In contrast, the uptake of natural PANC-1 EVs, B16–F10 EVs, and Hek-293 EVs in PANC-1 cells was studied *in vitro*, where the cells displayed preferential uptake of their daughter EVs over other EV types.^84^ Also, *in vivo* experiments showed that PANC-1 EVs demonstrated preferential accumulation in PANC-1 tumors compared to B16–F10 tumors. However, even in this case, tissue fluorescence postadministration was significantly higher in the liver, again pointing to a predominant liver accumulation of EVs.^84^

#### NP-Loaded EVs by Endogenous Strategies

Experiments from our own laboratory showed a remarkable selectivity *in vitro* of NP-loaded EVs using endogenous labeling.^36^ In this case, 40 nm Au nanoshells were incubated with the desired cells and the released Au-containing EVs were harvested. The Au load of the EVs enabled to obtain delivery selectivity with high accuracy through analysis of metal content. Selectivity experiments *in vitro* with EVs and cells from different origins clearly showed that EVs origin determined cell targeting properties and the differential uptake of therapeutic NPs.^36^ Then, the nanoshell-loaded EVs selectively mediated cell death *in vitro* mediated by photothermal (PT) effects.^36^ However, *in vivo* the selectivity of tumor delivery was considerably reduced.^87^ Post-mortem analyses indicated that around the 3% of the metal found in the animal was located in the tumor after intravenous injection in mice models, with the majority of Au retained in liver and spleen.^87^ In spite of this, tumor accumulation was still around 4 times higher than the content found with the same Au NPs after pegylation (EPR effect) and enough to cause tumor regression after NIR irradiation.^87^ Yong et al.^62^ introduced a biologically “self-assembled” EV loaded with porous silicon NPs (PSiNPs) that were first internalized by tumor cells and then exocytosed as EV-sheathed constructs. The authors observed enhanced tumor accumulation (∼2.5-fold versus free DOX and ∼2.3-fold versus DOX@PSiNPs at 24 h), improved extravasation and deeper parenchymal penetration.

In addition to parental cell lines, EVs from other cells with tumor tropism can be used for delivery. Wu et al.^63^ demonstrated that EV source influences distal barrier-crossing transport. ZnCdSe@ZnS core–shell QDs were endogenously encapsulated in EVs from HeLa (tumor) or J774A.1 (macrophage). They found that HeLa-derived exosome–QDs showed ∼2× higher delivery to neuronal cells after blood-brain barrier (BBB)-model transport compared to macrophage-derived exosome–QDs, highlighting that EV membrane composition can dominate trafficking outcomes. *In vivo*, QDs were injected in the tumor and inhibition of the EV secretion (GW4869) reduced brain deposition of QDs from ∼0.3% to ∼0.1% of the injected dose, providing quantitative support that EV are active transport mediators. Finally, other nontumoral cells can be used as endogenous sources of EVs with tumor tropism. Thus, Cu-containing carbon dots were incubated with mesenchymal stem cells (MSC) yielding CD-loaded EVs that produced a 40 fold increase in the efficiency of subsequent photodynamic therapy.^56^

#### NP-Loaded EVs by Exogenous Methods

Bolaños et al.^88^ loaded Au nanorods into EVs by passive incubation under gentle stirring and reported a high *in vitro* selectivity comparing the uptake of B16F10 (parental cells) and MLG cells. Biodistribution *in vivo* also gave encouraging results: metastatic lung nodules accumulation of injected NPs encapsulated in B16F10 EVs was 11 times higher than the NPs alone (Au mass was normalized by tissue weight). In spite of this, over 80% of injected dose was still found in the liver and spleen. Coextrusion has been widely used as a simple method to coat NPs with EVs membranes, thus, PB NPs were extruded together with U87-derived EVs to obtain PB-EVs hybrids.^72^ Interestingly, even this relatively crude coating method improved the NP properties and the authors reported an effective transit across the BBB with 4% accumulation in the brain region in comparison with pegylated PB NPs (1%) based on fluorescence measurements on extracted organs. A mixed ultrasonication-extrusion approach was used to cloak ZIF-8 and functional proteins with EVs from MDA-MB-231 human breast carcinoma cells.^77b^ The EV-Metal–Organic Framework (EV-MOF) hybrids exhibited 2 to 8-fold higher homotypic uptake across multiple cell lines while reducing opsonization and macrophage uptake (RAW264.7), leading to a high antitumor efficacy *in vivo*. Finally, hollow MnO_2_ NPs coextruded with HEK-293T EVs yielded a 2.4-fold tumor delivery gain in comparison with their uncoated counterparts while addressing hypoxia relief and GSH consumption under ultrasound irradiation thanks to their enzyme-mimicking activity.^71^

It is worth mentioning that the intrinsic targeting properties of EVs can be modified by adding functional groups. A good example is the modification of exosomes from MCF-7 cells with RGD peptide using a cell culture incubation approach.^57^ The RGD-containing EVs were harvested, and electroporation was used to load V_2_C NPs. RGD modification allowed targeting of the cell nucleus, increasing the efficiency of photothermal therapy (PTT) with NIR-II light. The authors reported a higher tumor accumulation (3.7 fold), reduced liver/spleen/kidney interception, and extended circulation half-time compared to naked V_2_C.

#### NP-Loaded EVs by *In Situ* Approaches

EVs loaded with metal NPs using the *in situ* loading strategy caused cell death selectively *in vitro* either through catalytic prodrug activation^58^ or through PtNP-mediated apoptosis,^59^ leveraging the selective tropism of the respective EVs. *In vivo*, they accumulated more efficiently in the tumoral xenograft tissues compared to pegylated NPs, enabling the activation of a prodrug supplied systemically. At the same time, a significantly lower accumulation occurred in the liver when PdNPs were delivered within the EVs (94.98% accumulation in the liver 48 h after administration of the pegylated vector, compared to 70.0% for PdNSs-loaded EVs).^74^ On the other hand, Pt NPs-loaded EVs were compared to systemic administration of cisplatin to treat a U251 xenograft tumor. *In vivo*, the amount of Pt in tumors doubled that found following the administration of the conventional drug at the same Pt doses and produced a strong decrease of tumor size while avoiding toxic effects observed in the animals undergoing cisplatin administration.^59^

#### Membrane-Coated EV and Artificial/Hybrid EVs

While obtaining functional biomimetic coatings of NPs using EVs membranes faces important challenges, as discussed above, it also has the key advantage of flexibility. There are no limitations regarding the nature of NPs (e.g., noble metals only for the CO reduction method) or their intrinsic toxicity (which would prevent using the natural endogenous method). We highlight a thorough study conducted by Zhang et al.^79^ spanning native EVs, EV–SPION NP biohybrids, and artificial EV-like vesicles. They developed human peripheral blood neutrophil-derived vesicular platforms to improve systemic tumor delivery by combining endogenous membrane functionality with magnetic guidance. Blood neutrophils were used either to (i) secrete EVs collected after 24 h in EV-depleted medium or (ii) generate reassembled neutrophil vesicles after serial extrusion yielding EV-like vesicles with scalable production. In an HGC27 xenograft model, SPION-decorated EVs under magnetic field guidance (EV-SPION/MF) produced the longest mean survival (86 days) with 40% of mice surviving to study end, outperforming PBS (45 days), SPION alone (47 days), EVs (61 days), and EV-SPION without MF (59 days), thereby quantitatively linking EV interface engineering to survival benefit. Recently, hybrid EVs formed by breakable organosilica NPs coated with B16–F10 EV-derived membranes were preferentially internalized *in vitro* by their parental cells (65.1%) compared to NIH-3T3 cells (45.2%) after 48 h of incubation. When tested *in vivo*, a significant amount of the NPs coated with the EV-membranes accumulated successfully in the tumoral regions (in particular, in the metastatic model of melanoma).^62,89^

In an interesting microfluidic-mediated approach, conjugation with cholesterol-modified aptamers improved the targeting properties of PLGA NPs coated with tumor-derived EVs.^77c^ Aptamer modification favored specific binding to nucleolin on tumor cell membranes, thereby boosting tumor localization beyond homotypic targeting. In an MDA-MB-231 xenograft model, this strategy translated into higher tumor delivery, achieving roughly 1.5-fold higher tumor signal than nonaptamer EV-PLGA and random-aptamer controls, respectively, and a 12.5-fold increase versus a lipid-coated control. This again highlights the improvement on targeting capabilities that can be obtained by suitable modifications of EV membranes.

Developing functional artificial or hybrid (i.e., liposome-EV) membranes able to mimic the roles of natural EVs requires identification of the key factors that provide the desired functionality to EVs. In an interesting example of efforts to develop artificial membranes, SiO_2_ NPs were encapsulated within phospholipid-based artificial vesicles.^80^ Computational modeling was used to design lipidic layers, taking into account lipidomic data of prostate cancer-derived EVs. In the case of hybrid membranes, a recent study by Abdel-Bar et al.^90^ demonstrated the advantage of combining EVs from 4T1 and B16F10 cells with liposome NPs. The EV hybrids outperformed the liposome NPs and exhibited preferential association with their parental tumor cell types in both 4T1 and B16F10 tumor models.

#### *In Vivo* Studies: Problems and Possible Artifacts

In the last 10 years, *in vitro* and *in vivo* EV-based delivery of NPs has been explored intensely. Especially *in vivo*, the comparison among the results of different studies is frequently obscured by a lack of basic data relevant to the study. For instance, it is often unclear whether the administered dose comprises only EV-encapsulated NPs or if they are accompanied by a significant proportion of free (unassociated) NPs and/or unloaded EVs. Similarly, EV heterogeneity can be high.^91^ A standardized EV isolation strategy is still lacking, and the amount of isolated EVs is often low. In addition, a large variability of sizes and the presence of different populations of co-isolated EVs can be suspected since the purity and type of EVs employed in a study are rarely reported. Even more important, especially for the case of EV-NP hybrids, encapsulation methodologies are often aggressive, likely causing the disruption of the membrane. This may not only affect intrinsic targeting properties but also likely enhance recognition and removal by the MPS system.^42,45^

With these caveats, Table 1 gathers a selection of studies that provide a glimpse of the varied research landscape in this area. The origin of the EVs and type of NP used, the encapsulation method, the type of tumor cells and animal model used, and finally the administration route are specified to describe the diverse scenarios studied. Then, the next two columns describe the method used to measure accumulation and the time of measurement. The efficiency of the delivery is described in the next three columns, namely, the comparison to EPR-based delivery, where available, the tumor versus liver accumulation, and finally, the therapeutic outcome. Deliberately, we have avoided reporting on biodistribution data, in spite of the fact that they are, together with therapeutic outcomes, the main result of these investigations. The reason is the difficulty of comparing results among different studies because of the divergent criteria employed to report accumulation in different tissues and organs. In many cases, the amount of EV-NP hybrids reaching the different organs is not accurately determined, just reported as relative intensity of optical signals, with all the associated problems (see below). When a more accurate determination is attempted (e.g., by *ex vivo* chemical analysis), biodistribution is often reported as relative amounts in the different organs (rather than as percentage over the total dose injected) or as specific concentrations (mg of NP per mg of organ) without reference to the weights of each organ, which makes it impossible to calculate the absolute delivery efficiency.

**Table 1:** Selection of *In Vivo* Studies Using EV-NPs for Therapy

EVs origin	NPs type	Encapsulation strategy	Target cell/tissue	Animal model	Administration route	Time point analysis	Measurement method	Comparison with EPR based delivery	Comparison of accumulation indicators in tumor and liver	Therapeutic outcome	Notes	ref
Human placental MSCs (hpMSCs).	Cu-doped carbon dots	Endogenous biogenesis pathway	U87-MG, A549, U251-MG	U87-MG xenograft mode	Intravenous	24 h after injection	Fluorescence *ex vivo* by IVIS due to intrinsic fluorescence emission of carbon dots	*Not provided*	*Not provided*	Significant tumor reduction	–^a^	^56^
MCF-7 breast cancer cells, engineered with RGD by incubating donor cells with DSPE–PEG–RGD prior to isolation	Vanadium carbide (V_2_C) quantum dots (MXene QDs) functionalized with PEG and TAT (nuclear localization)	Loaded into EV-RGD by electroporation (200 V, 100 μF)	MCF-7 (with NHDF as normal-cell control; also A549 used for toxicity)	MCF-7 xenografts under 1064 nm irradiation	Intravenous	24 h after injection	Fluorescence from FAM-labeled MXene QDs and DiI-labeled EVs	2.11-fold higher accumulation of encapsulated MXene QDs compared with PEGylated MXene QDs	≈ 9% ID/g in tumor vs ≈ 16% ID/g in liver	Strong tumor reduction	–^a^	^57^
U251-MG	Ultrasmall Pt NPs	CO reduction *in situ*	U251-MG	Xenograft U251-MG tumor bearing nude mice	Intravenous	24 h after injection	ICP-MS analysis of Pt	*Not provided (Pt NPs too small for EPR, renal excretion likely)*	≈0.35% of the injected dose in tumor vs ≈2% of the injected dose in liver	Strong tumor reduction	–^a^	^59^
MCF-7, MDA-MB-231, and 4T1 cultures.	Magnetic mesoporous silica nNPs(Fe_3_O_4_@mSiO_2_) functionalized with glucose oxidase (GOx)	Ultrasonication + vortex mixing	Homotypic tumor cells (MCF-7, MDA-MB-231, 4T1	4T1 subcutaneous breast tumor model (BALB/c mice)	Intravenous	3, 6, 12, 24, 48 h after injection	Fluorescence *ex vivo*	*Not provided*	*Not provided*	Significant tumor reduction		^69^
HEK-293T (human embryonic kidney	Hollow MnO_2_ nanoparticles coloaded with ICG (sonosensitizer) and FX11 (glycolysis inhibitor)	Sonication and extrusion (12x)	MCF-7 human breast cancer (focus on hypoxic tumors)	MCF-7 tumor xenograft mice	Intravenous	24 h after injection	Fluorescence *ex vivo* by IVIS	≈16% of ID/g when encapsulated in EVs vs ≈8% of ID/g of free M (ICG/FX11)	≈16% of ID/g in tumor vs of ID/g 20% of ID/g in the liver	Strong tumor reduction	–^a^	^71^
U-87	Prussian Blue NPs	Serial extrusion through 400/200/100 nm membranes (11 passes each)	U-87	Subcutaneous U-87 model	Intravenous	24 h after injection	Fluorescence *ex vivo* by IVIS of DiD labeled EVs and ICP-MS of Fe	*Not provided*	≈4 × 10^8^ of fluorescence intensity (a.u.) in tumor vs ≈6 × 10^9^ of fluorescence intensity (a.u.) in liver	Strong tumor reduction	–^a^	^72^
				Orthotopic U-87-luc brain tumor model								
A549	Pd NSs	CO reduction *in situ*	A549	Xenograft A549 tumor bearing nude mice	Intravenous	48 h after injection	ICP-MS analysis of Pd	2.25% of the detected dose of Pd-EVs (2.5 times higher) vs 0.91% of the detected dose of Pd-NPs after 48 h	2.25% of the detected dose vs 94.98% of the detected dose of Pd-NPs in liver	Significant tumor reduction		^74^
MDA-MB-231	ZIF-8 (Zn^2+^/2-methylimidazole) MOF nanoparticles encapsulating biofunctional proteins	Ultrasonication and repeated extrusion.	MDA-MB-231231 vs 293*T*/3T3/CAD/MCF7/SH-SY5Y	Orthotopic MDA-MB-231 tumor-bearing mice	Intravenous	72 h after injection	Fluorescence *ex vivo* by IVIS	4-fold higher accumulation of encapsulated MOFs compared with naked MOFs	≈2 × 10^4^ of fluorescence intensity (a.u.) vs ≈0.9 × 10^4^ of fluorescence intensity in the liver	Strong tumor reduction	–^a^	^77b^
MDA-MB-231	PLGA	Microfluidic sonication post modification with cholesterol modified AS1411 aptamers	MDA-MB-231 tumors/cells RAW 264.7 macrophages	MDA-MB-231 xenograft tumor–bearing NOD SCID mice	Intravenous	48 h after injection	Fluorescence ex vivo by IVIS	12.5-fold higher compared with AS1411-modified lipid-PLGA NPs	≈1% of ID/g in tumor vs of ID/g 20% of ID/g in the liver	*Not provided*	–^a^	^77c^
Primary human neutrophils	SPION surface decoration with transferrin	Incubation and magnetic collection	HGC27 (gastric cancer)	HGC27 subcutaneous xenografts in BALB/c nude mice	Intravenous	72 h after injection	Fluorescence *ex vivo* by IVIS (DiR labeled EVs)	*Not quantitatively compared with EPR-based delivery*	*Quantitative data in the liver not provided*	Significant tumor reduction	–^a^	^79^
hpMSCs	Au NPs	Incubation of NPs in hpMSCs cell culture	SKOV_Luc	Multinodular model with SKOV_Luc cells in nude mice	Intravenous	72 h after injection	ICP-MS of Au	5.13% of detected dose (in tumor areas) when encapsulated in EVs vs 3.29% of detected dose (in tumor areas) of PEGylated NPs	35.13% of detected dose in tumor areas, respectively vs 90.82% of detected dose in the liver.	Strong tumor reduction	–^b^	^87^
B16–F10	Breakable SiO_2_ NPs	Coating with EVs membranes	B16–F10	Metastatic model B16–F10	Intravenous	4 h after injection	Fluorescence *ex vivo* by IVIS (ICP-OES of Si is also provided)	≈6 × 10^13^ radiant efficiency when encapsulated in EVs vs ≈5 × 10^12^ radiant efficiency when administered naked NPs.	≈6 × 10^13^ radiant efficiency in the lungs with metastasis vs ≈4 × 10^13^ radiant efficiency in the liver	Significant tumor reduction	–^a^ ^,^ ^b^	^89^
Natural grapefruit EVs	Doxorubicin-loaded heparin-based NPs	Surface modification of EVs	Glioma cells (LN229, U251 and U87)	Glioma-bearing nude mice	Intravenous	96 h after injection	*Ex vivo* Cy7 fluorescence signal	≈4 × 10^6^ average fluorescence signal when encapsulated in EVs vs ≈0.5 × 10^6^ average fluorescence signal of naked NPs.	≈4 × 10^6^ average fluorescence signal in tumor vs ≈10 × 10^6^ average fluorescence signal in liver.	Strong tumor reduction	–^a^	^97^
Tumor-associated macrophages (Raw 264.7)	SPIONs	Electroporation	Glioma (U251 cells)	Glioma-bearing nude mice	Intravenous	–	*Not provided*	*Not provided*	*Not provided*	Strong tumor reduction	–^c^	^98^
Scamous cell cells carcinoma (SCC9)	Gold NPs	Simple incubation	SCC9 cells	Tongue squamous cell carcinoma (TSCC)	Intratumoral	–	*Not provided*	*Not provided*	*Not provided*	Strong tumor reduction	–^c^	^99^
Advanced hepatocellular carcinoma (HCC)	sequential nanocatalysts GOD-ESIONs@EVs	Covalently bound surface engineering	Huh 7	Huh 7 tumor-bearing nude mice	Intravenous	4 h after injection	*Ex vivo* Cy5.5 fluorescence signal by Maestro system	≈300 au relative fluorescence value when encapsulated in EVs vs ≈100 au relative fluorescence value of Pegylated NPs.	≈300 au relative fluorescence value in tumor vs ≈250 au relative fluorescence value in the liver	Strong tumor reduction	–^a^	^100^
HCC Huh 7												

a Data reported were read from the manuscript figures (numerical data were not provided in the original manuscript).

b Data for a xenograft model were also provided in the reference.

c Nonquantitative or semiquantitative data were provided in the original manuscript.

There are also problems related to the labels used to monitor the *in vivo* biodistribution of EVs. Different fluorophores (e.g., fluorescent dyes DiR or PKH probes) can be used to label EV membranes, allowing tracking their accumulation by measurements carried out either *in vivo* or *ex vivo*, in images of the different organs.^42^ However, there are many problems regarding optical labeling, starting with their resolution for different tissue depths, and the artifacts that can result from the labeling molecules themselves (sometimes they can be released from EVs, leading to false signals, they may favor EVs aggregation and they could also interfere with EVs membrane and surface proteins, modifying their natural targeting and immune evading capacity).^92^ The use of MRI-sensitive labels such as PERFECTA, a branched molecule with 36 magnetically equivalent ^19^F atoms,^93^ represents an interesting alternative for *in vivo* imaging, since MRI could in principle give a higher resolution and precision compared to optical tagging. However, labeling with PERFECTA also raises similar questions to those discussed for optical tags regarding its effect on EVs membrane and surface proteins.

A different approach to obtain EVs biodistribution is their labeling with metallic NPs and their subsequent detection by Inductively Coupled Plasma (ICP) analysis. Although this methodology is highly specific and sensitive (and can yield rather different results from optical labeling^87^), it is not exempt from problems. First, it gives biodistribution only at one time point, not allowing real-time tracking of the biodistribution. In addition, depending on the method used to label EVs with metal NPs (e.g., natural incubation and harvesting of released EVs vs electroporation), the effects on the EVs membranes can be very different. Finally, the specific load (amount of metal per EV) can be heterogeneous, raising questions regarding their behavior *in vivo* as a function of the loading. Even for methods that guarantee homogeneity of loading among EVs and seem to preserve their membrane functionality (such as reduction of metal precursors with CO), the presence of metallic NPs could change the natural rigidity and/or mechanical properties of EVs, potentially leading to a different behavior *in vivo*. Finally, there is still limited evidence and no consensus among the EVs community regarding how the EVs storage, stability, administration route, or the type of animal model employed affect EVs behavior *in vivo*.

This overview highlights the main difficulties and roadblocks in current research using EVs as NP delivery vectors. In spite of this, the evidence gathered is enough to support the outstanding role of EVs as NP delivery vectors (see Table 1), with better selectivity and immune evasion capabilities compared to naked, pegylated, or even target-functionalized NPs. EV-NP hybrids offer the possibility of finally breaking the 1% delivery barrier in cancer nanomedicine. However, difficulties on the way should not be underestimated. The starting point is the recognition of a sobering fact: despite the high selectivity of EV-mediated NP delivery *in vitro*, targeting yields drop significantly *in vivo* (even in favorable xenograft models using immunodeficient animals).

Many EV-based therapies are being clinically tested. In 2023, more than 40 EV-based clinical trials were registered for the treatment of COVID-19, Crohn’s disease, and diabetes.^94^ However, to date no approved engineered EV-based cancer therapies exist.^95^ This can be attributed in part to unsolved challenges, including Good manufacturing practice (GMP)-compliant production, reproducibility, purification yield, safety concerns or side effects associated with EV-based therapies.^96^ But other, more important problems, relate to knowledge gaps regarding EVs properties and their behavior *in vivo*. Below we list some of the main concerns and research needs to progress in this field.

- The natural (endogenous) incorporation of NPs probably appears as the best option for preserving pristine EVs properties. However, it is inherently inefficient and poorly controllable. NP modification (e.g., by functionalization or coating) may help to alleviate the problems related to inhomogeneity of loading or intrinsic NP toxicity. In addition, the EV-producing cells can be engineered to stimulate EVs production or to produce EVs with desirable features on their surface (e.g., enhanced expression of “self” markers that reduce MPS uptake).
- Exogenous methods such as sonication and electroporation need to be fine-tuned to minimize EV damage. New device designs are needed to precisely control field intensity, residence time, and mode of contact between EVs and NPs. Also, methods that are in principle friendlier toward EV membranes, such as *in situ* synthesis, need to be extended beyond noble metals.
- A wide assessment of the different loading methods is required, ideally relating EVs characteristics (e.g., type, size, origin) with particle properties (size, charge, nature), leading to a set of practical rules that allow a rational selection of the loading method, depending on the specific scenario.
- Similarly, the selection of EVs isolation method (e.g., ultracentrifugation, size-exclusion chromatography, tangential flow filtration, etc.) previous to NP loading, and of the postloading purification strategy (to remove unencapsulated NPs, unloaded EVs and debris) needs to consider NPs characteristics. For instance, ultracentrifugation after loading needs to be tuned to allow for the different properties of loaded EVs.
- A clear lack of knowledge exists regarding the internalization mechanisms of EV-NP hybrids. The relative contribution of different internalization routes (e.g., receptor-mediated internalization vs membrane fusion) needs to be ascertained and related to the properties of EV-NP hybrid, internalization conditions, and target cells. Also, the methods to measure *in vitro* the selectivity in the delivery toward different cell types require standardization. Chemical analysis-based methods are more accurate than those based on fluorescent labeling and less prone to artifacts.
- Biodistribution studies with EV-NP hybrids are the cornerstone to measure delivery efficiency *in vivo* and provide key information (liver and spleen retention, circulation time, tumor accumulation) to improve successive generations of delivery vectors. New labeling methods for NP-containing EVs are sorely needed. Verifying that labeling does not interfere with targeting and does not produce artifacts or significantly alter the properties (e.g., hydrodynamic size, agglomeration) of EV-NP hybrids is essential.
- There is still a lack of information on the BC formed around EVs in different biological environments. This corona determines their new biological identity, and it is reasonable to assume that it has a strong influence on EV targeting and immune system recognition. Research in this area is urgently needed, also to ascertain how the association of EVs with NPs will modify the BC properties.
- Finally, up to now *in vivo* studies using EV-NPs hybrids have mainly employed administration by intravenous injection in discrete shots (bolus effect). Other delivery routes (e.g., inhaled, intranasal, intraperitoneal) and administration schemes (e.g., gradual administration, pretreatment of macrophages with other NPs) should be explored, as they will give rise to different biodistributions and open up new pathways for tumor targeting.

## References

[1] Global Cancer Observatory . https://gco.iarc.fr/en April 2026.

[2] Global cancer burden growing, amidst mounting need for services. https://www.who.int/news/item/01-02-2024-global-cancer-burden-growing--amidst-mounting-need-for-services April 2026. PMC11115397 38438207

[3] He H., Liu L., Morin E. E., Liu M., Schwendeman A. (2019). Survey of Clinical Translation of Cancer Nanomedicines-Lessons
Learned from Successes and Failures. Acc. Chem.
Res..

[4] Wilhelm S., Tavares A. J., Dai Q., Ohta S., Audet J., Dvorak H. F., Chan W. C. W. (2016). Analysis of nanoparticle delivery
to tumous. Nat. Rev. Mater..

[5] Cheng Y.-H., He C., Riviere J. E., Monteiro-Riviere N. A., Lin Z. (2020). Meta-Analysis of Nanoparticle
Delivery to Tumors Using a Physiologically Based Pharmacokinetic Modeling
and Simulation Approach. ACS Nano.

[6] Choi C. H. J., Alabi C. A., Webster P., Davis M. E. (2010). Mechanism
of active targeting in solid tumors with transferrin-containing gold
nanoparticles. Proc. Natl. Acad. Sci. U. S.
A..

[7] Dai Q. (2018). Quantifying the Ligand-Coated Nanoparticle Delivery to Cancer Cells
in Solid Tumors. ACS Nano.

[8] Kisby T., Yilmazer A., Kostarelos K. (2021). Reasons for
success and lessons learnt
from nanoscale vaccines against COVID-19. Nat.
Nanotechnol..

[9] Nano . The magazine for small science. https://nano-magazine.com/news/2023/9/13/ahead-in-nanotechnology-funding-predictions-and-evolving-trends. April 2026.

[10] Bhatia S. N., Chen X., Dobrovolskaia M. A., Lammers T. (2022). Cancer nanomedicine. Nat. Rev.
Cancer.

[11] Gustafson H. H., Holt-Casper D., Grainger D. W., Ghandehari H. (2015). Nanoparticle
Uptake: The Phagocyte
Problem. Nano Today.

[12] Yoon Y. J. (2013). Three-Dimensional Imaging
of Hepatic Sinusoids in Mice Using Synchrotron
Radiation Micro-Computed Tomography. PLoS One.

[13] Mak K. M., Shin D. W. (2021). Hepatic sinusoids
versus central veins: Structures,
markers, angiocrines, and roles in liver regeneration and homeostasis. Anat. Rec..

[14] Tsoi K. M. (2016). Mechanism of hard-nanomaterial clearance by the liver. Nat. Mater..

[15] Wang L., Quine S., Frickenstein A. N., Lee M., Yang W., Sheth V. M., Bourlon M. D., He Y., Lyu S., Garcia-Contreras L., Zhao Y. D., Wilhelm S. (2024). Exploring and Analyzing
the Systemic Delivery Barriers for Nanoparticles. Adv. Funct. Mater..

[16] Jalil A. R., Tobin M. P., Discher D. E. (2022). Suppressing
or Enhancing Macrophage Engulfment through the Use of CD47 and Related
Peptides. Bioconjug Chem..

[17] Liu T., Choi H., Zhou R., Chen I.-W. (2015). RES blockade: A
strategy for boosting efficiency of
nanoparticle drug. Nano Today.

[18] Poon W. (2019). Elimination Pathways
of Nanoparticles. ACS
Nano.

[19] Fan D., Cao Y., Cao M., Wang Y., Cao Y., Gong T. (2023). Nanomedicine
in cancer therapy. Signal Transduct. Target.
Ther..

[20] Menon I., Zaroudi M., Zhang Y., Aisenbrey E., Hui L. (2022). Fabrication of active targeting lipid
nanoparticles: Challenges and
perspectives. Mater. Today Adv..

[21] Stefanick J. F., Omstead D. T., Kiziltepe T., Bilgicer B. (2019). Dual-receptor targeted
strategy in nanoparticle design achieves tumor cell selectivity through
cooperativity. Nanoscale.

[22] Salvati A. (2013). Transferrin-functionalized
nanoparticles lose their targeting capabilities
when a biomolecule corona adsorbs on the surface. Nat. Nanotechnol..

[23] Wang W., Mu S., Zhang J., Shan G., Li X., Wang R., Guo T., He X. (2026). Trojan Horse Strategy: How Biomimetic Nanomedicine Remodels the Tumor
Microenvironment. Adv. Sci..

[24] Areberg J. (1999). Gamma camera imaging
of platinum in tumours and tissues of patients
after administration of 191Pt-cisplatin. Acta
Oncol..

[25] Dawidczyk C. M., Russell L. M., Hultz M., Searson P. C. (2017). Tumor accumulation
of liposomal doxorubicin in three murine models: Optimizing delivery
efficiency. Nanomedicine.

[26] Obata H. (2022). Precise quantitative
evaluation of pharmacokinetics of cisplatin
using a radio-platinum tracer in tumor-bearing mice. Nucl. Med. Commun..

[27] van
Niel G., D’Angelo G., Raposo G. (2018). Shedding light on the
cell biology of extracellular vesicles. Nat.
Rev. Mol. Cell Biol..

[28] Cheng L., Hill A. F. (2022). Therapeutically
harnessing extracellular vesicles. Nat. Rev.
Drug Discovery.

[29] Jeppesen D. K., Fenix A. M., Franklin J. L., Higginbotham J. N., Zhang Q., Zimmerman L. J., Liebler D. C., Ping J., Liu Q., Evans R., Fissell W. H., Patton J. G., Rome L. H., Burnette D. T., Coffey R. J. (2019). Reassessment of Exosome Composition. Cell.

[30] Welsh J. A. (2024). Minimal information for studies of extracellular vesicles (MISEV2023):
From basic to advanced approaches. J. Extracell.
Vesicles.

[31] Seibold T., Waldenmaier M., Seufferlein T., Eiseler T. (2021). Small Extracellular
Vesicles and Metastasis—Blame the Messenger. Cancers.

[32] van
Niel G. (2022). Challenges and directions in studying cell–cell
communication by extracellular vesicles. Nat.
Rev. Mol. Cell Biol..

[33] Nawaz M. (2018). Extracellular Vesicles and Matrix Remodeling Enzymes:
The Emerging
Roles in Extracellular Matrix Remodeling, Progression of Diseases
and Tissue Repair. Cells.

[34] Chen C. C. (2016). Elucidation of Exosome Migration across the Blood-Brain Barrier Model
In Vitro. Cell. Mol. Bioeng..

[35] Lin Y., Zhang C., Xiang P., Shen J., Sun W., Yu H. (2020). Exosomes derived from
HeLa cells break down vascular integrity by triggering endoplasmic
reticulum stress in endothelial cells. J. Extracell.
Vesicles.

[36] Sancho-Albero M., Navascues N., Mendoza G., Sebastian V., Arruebo M., Martin-Duque P., Santamaria J. (2019). Exosome origin determines cell targeting and
the transfer of therapeutic
nanoparticles towards target cells. J. Nanobiotechnology.

[37] Mathieu M., Martin-Jaular L., Lavieu G., Théry C. (2019). Specificities
of secretion and uptake of exosomes and other extracellular vesicles
for cell-to-cell communication. Nat. Cell Biol..

[38] Hoshino A. (2015). Tumour exosome integrins
determine organotropic metastasis. Nature.

[39] Zhang S., Liao X., Chen S., Qian W., Li M., Xu Y., Yang M., Li X., Mo S., Tang M., Wu X., Hu Y., Li Z., Yu R., Abudourousuli A., Song L., Li J. (2022). Large Oncosome-Loaded
VAPA Promotes Bone-Tropic Metastasis of Hepatocellular Carcinoma Via
Formation of Osteoclastic Pre-Metastatic Niche. Adv. Sci..

[40] Keller S. (2007). CD24 is a marker of
exosomes secreted into urine and amniotic fluid. Kidney Int..

[41] Belhadj Z., He B., Deng H., Song S., Zhang H., Wang X., Dai W., Zhang Q. (2020). A combined ‘eat me/don’t eat me’ strategy based
on extracellular vesicles for anticancer nanomedicine. J. Extracell. Vesicles.

[42] Chaudhari A. P. (2026). The status of extracellular
vesicles as drug carriers and therapeutics. Nat. Rev. Bioeng..

[43] Wiklander O. P. B., Nordin J. Z., O'Loughlin A., Gustafsson Y., Corso G., Mager I., Vader P., Lee Y., Sork H., Seow Y., Heldring N., Alvarez-Erviti L., Smith C. E., Le Blanc K., Macchiarini P., Jungebluth P., Wood M. J. A., Andaloussi S. E. (2015). Extracellular
vesicle in vivo biodistribution is determined by cell source, route
of administration and targeting. J. Extracell.
Vesicles.

[44] Takov K., Yellon D. M., Davidson S. M. (2017). Confounding
factors in vesicle uptake
studies using fluorescent lipophilic membrane dyes. J. Extracell. Vesicles.

[45] Kang M., Jordan V., Blenkiron C., Chamley L. W. (2021). Biodistribution
of extracellular vesicles following administration into animals: A
systematic review. J. Extracell. Vesicles.

[46] Bonsergent E., Grisard E., Buchrieser J., Schwartz O., Thery C., Lavieu G. (2021). Quantitative characterization
of extracellular vesicle
uptake and content delivery within mammalian cells. Nat. Commun..

[47] Mulcahy L. A., Pink R. C., Carter D. R. F. (2014). Routes
and mechanisms of extracellular
vesicle uptake. J. Extracell. Vesicles.

[48] Edgar J. R., Manna P. T., Nishimura S., Banting G., Robinson M. S. (2016). Tetherin
is an exosomal tether. Elife.

[49] Prada I. (2016). A new approach to follow a single extracellular vesicle-cell interaction
using optical tweezers. Biotechniques.

[50] Charoenviriyakul C., Takahashi Y., Morishita M., Nishikawa M., Takakura Y. (2018). Role of Extracellular
Vesicle Surface Proteins in the
Pharmacokinetics of Extracellular Vesicles. Mol. Pharmaceutics.

[51] Ezzat K. (2019). The viral protein corona
directs viral pathogenesis and amyloid aggregation. Nat. Commun..

[52] Nolte-'t
Hoen E., Cremer T., Gallo R. C., Margolis L. B. (2016). Extracellular vesicles
and viruses: Are they close relatives?. Proc.
Natl. Acad. Sci. U. S. A..

[53] Musicò A. (2024). Extracellular vesicles
of different cellular origin feature distinct biomolecular corona
dynamics. Nanoscale Horizons.

[54] Wolf M. (2022). A functional corona
around extracellular vesicles enhances angiogenesis,
skin regeneration and immunomodulation. J. Extracell.
Vesicles.

[55] Sancho-Albero M. (2019). Efficient encapsulation
of theranostic nanoparticles in cell-derived
exosomes: leveraging the exosomal biogenesis pathway to obtain hollow
gold nanoparticle-hybrids. Nanoscale.

[56] Besbinar O. (2025). Cell-Driven Encapsulation
of Chlorophyllin-Based Carbon Dots within
Exosomes for Enhanced Photodynamic Therapy: miRNA Profiling Reveals
Mechanistic Insights. ACS Appl. Mater. Interfaces.

[57] Cao Y., Wu T., Zhang K., Meng X., Dai W., Wang D., Dong H., Zhang X. (2019). Engineered Exosome-Mediated Near-Infrared-II
Region V(2)C Quantum Dot Delivery for Nucleus-Target Low-Temperature
Photothermal Therapy. ACS Nano.

[58] Sancho-Albero M. (2019). Cancer-derived exosomes
loaded with ultrathin palladium nanosheets
for targeted bioorthogonal catalysis. Nat. Catal..

[59] Sancho-Albero M., Martin-Pardillos A., Lujan L., Sebastian V., Santamaria J., Martin-Duque P. (2022). Exosomes loaded with ultrasmall Pt
nanoparticles: a novel low-toxicity alternative to cisplatin. J. Nanobiotechnology.

[60] Zickler A. M. (2024). Novel Endogenous Engineering Platform for Robust Loading and Delivery
of Functional mRNA by Extracellular Vesicles. Adv. Sci..

[61] Alhasan A. H., Patel P. C., Choi C. H. J., Mirkin C. A. (2014). Exosome Encased
Spherical Nucleic Acid Gold Nanoparticle Conjugates as Potent MicroRNA
Regulation Agents. Small.

[62] Yong T., Zhang X., Bie N., Zhang H., Zhang X., Li F., Hakeem A., Hu J., Gan L., Santos H. A., Yang X. (2019). Tumor exosome-based
nanoparticles are efficient drug carriers for
chemotherapy. Nat. Commun..

[63] Wu D. (2024). Exosome Heterogeneity Affects the Distal ‘Barrier-Crossing’
Trafficking of Exosome Encapsulated Quantum Dots. ACS Nano.

[64] Betzer O. (2017). In Vivo Neuroimaging of Exosomes Using Gold Nanoparticles. ACS Nano.

[65] Qian Z. (2020). A moisturizing chitosan-silk fibroin dressing with silver nanoparticles-adsorbed
exosomes for repairing infected wounds. J. Mater.
Chem. B.

[66] Luan X. (2017). Engineering exosomes
as refined biological nanoplatforms for drug
delivery. Acta Pharmacol. Sin..

[67] Hood J. L., Scott M. J., Wickline S. A. (2014). Maximizing
exosome colloidal stability
following electroporation. Anal. Biochem..

[68] Wu T., Liu Y., Cao Y., Liu Z. (2022). Engineering Macrophage Exosome Disguised
Biodegradable Nanoplatform for Enhanced Sonodynamic Therapy of Glioblastoma. Adv. Mater..

[69] Li M., Tai Q., Shen S., Gao M., Zhang X. (2024). Biomimetic Exosome-Sheathed
Magnetic Mesoporous Anchor with Modification of Glucose Oxidase for
Synergistic Targeting and Starving Tumor Cells. ACS Appl. Mater. Interfaces.

[70] Khongkow M., Yata T., Boonrungsiman S., Ruktanonchai U. R., Graham D., Namdee K. (2019). Surface modification
of gold nanoparticles with neuron-targeted exosome for enhanced blood–brain
barrier penetration. Sci. Rep..

[71] Truong
Hoang Q. (2023). Exosome membrane-sheathed and multi-stimuli-responsive
MnO2 nanoparticles with self-oxygenation and energy depletion abilities
potentiate the sonodynamic therapy of hypoxic tumors. Chem. Eng. J..

[72] Hill M. L., Chung S.-J., Woo H.-J., Park C. R., Hadrick K., Nafiujjaman M., Kumar P. P. P., Mwangi L., Parikh R., Kim T. (2024). Exosome-Coated Prussian Blue Nanoparticles for Specific Targeting
and Treatment of Glioblastoma. ACS Appl. Mater.
Interfaces.

[73] Sebastian V. (2021). Nondestructive production of exosomes loaded with ultrathin palladium
nanosheets for targeted bio-orthogonal catalysis. Nat. Protoc..

[74] Sancho-Albero M. (2024). Extracellular Vesicles-Mediated Bio-Orthogonal Catalysis in Growing
Tumors. Cells.

[75] Li X., He S., Luo B., Li P., Chen X., Wu M., Song C., Liu C., Yang T., Zhang X., Yang X., Hu J. (2023). Engineered Extracellular Vesicles
to Enhance Antigen Presentation for Boosting Light-Driven Tumor Immunotherapy. Small.

[76] Chen Y., Douanne N., Wu T., Kaur I., Tsering T., Erzingatzian A., Nadeau A., Juncker D., Nerguizian V., Burnier J. V. (2025). Leveraging nature’s nanocarriers: Translating
insights from extracellular vesicles to biomimetic synthetic vesicles
for biomedical applications. Sci. Adv..

[77] Liu C. (2019). Microfluidic Sonication
to Assemble Exosome Membrane-Coated Nanoparticles for Immune Evasion-Mediated
Targeting. Nano Lett..

[78] Lee J.-R., Park B.-W., Kim J., Choo Y. W., Kim H. Y., Yoon J.-K., Kim H., Hwang J.-W., Kang M., Kwon S. P., Song S. Y., Ko I. O., Park J.-A., Ban K., Hyeon T., Park H.-J., Kim B.-S. (2020). Nanovesicles derived
from iron oxide nanoparticles-incorporated mesenchymal stem cells
for cardiac repair. Sci. Adv..

[79] Zhang J., Ji C., Zhang H., Shi H., Mao F., Qian H., Xu W., Wang D., Pan J., Fang X., Santos H. A., Zhang X. (2022). Engineered neutrophil-derived
exosome-like vesicles for targeted
cancer therapy. Sci. Adv..

[80] Rosso G. (2025). Rational Design of EV-Mimicking Nanoparticles with Polarity-Based
Recognition Potential for Advanced Nanocarrier Development. ACS Appl. Nano Mater..

[81] Zhang J. (2025). Comparison of the Cytotoxicity, Internalization and Anti-Cancer Drug
Delivery Efficacy of Nature Killer Cell Derived Nanovesicles and Extracellular
Vesicles. Int. J. Nanomedicine.

[82] Shao M. (2023). Exosome membrane-coated
nanosystems: Exploring biomedical applications
in cancer diagnosis and therapy. Matter.

[83] Cardellini J., Normak K., Gerlt M., Makasewicz K., Seiffert C., Capasso Palmiero U., Ye S., Gonzalez
Gomez M. A., Pinero Y., Rivas J., Bongiovanni A., Bergese P., Arosio P. (2025). Microfluidics-Driven Manufacturing
and Multiscale Analytical Characterization of Nanoparticle-Vesicle
Hybrids. Adv. Healthc. Mater..

[84] Xu L., Faruqu F. N., Liam-or R., Abu Abed O., Li D., Venner K., Errington R. J, Summers H., Wang J. T.-W., Al-Jamal K. T. (2020). Design of experiment
(DoE)-driven in vitro and in vivo
uptake studies of exosomes for pancreatic cancer delivery enabled
by copper-free click chemistry-based labelling. J. Extracell. Vesicles.

[85] Kim S. M. (2017). Cancer-derived
exosomes as a delivery platform of CRISPR/Cas9 confer
cancer cell tropism-dependent targeting. J.
Controlled Release.

[86] Emam S. E. (2019). Cancer cell-type tropism is one of crucial determinants
for the efficient
systemic delivery of cancer cell-derived exosomes to tumor tissues. Eur. J. Pharm. Biopharm..

[87] Sancho-Albero M., Encinas-Gimenez M., Sebastian V., Perez E., Lujan L., Santamaria J., Martin-Duque P. (2022). Transfer of photothermal nanoparticles
using stem cell derived small extracellular vesicles for in vivo treatment
of primary and multinodular tumours. J. Extracell.
Vesicles.

[88] Bolaños K. (2025). In vivo Targeted Delivery
of Extracellular Vesicle–Gold Nanorod
Hybrids to Metastatic Melanoma Lung Tumors. Int. J. Nanomedicine.

[89] Sancho-Albero M. (2025). Melanoma extracellular vesicles membrane coated nanoparticles as
targeted delivery carriers for tumor and lungs. Mater. Today Bio.

[90] Abdel-Bar H. M. (2025). Optimizing Exosome Lipid Hybrid Nanoparticles for Enhanced siRNA
Delivery and Improved Therapeutic Anticancer Efficacy In Vivo. ACS Nano.

[91] Panda S. S. (2026). Exosomal heterogeneity and functional zonation in cancer drug resistance. Drug Discovery Today.

[92] Fortunato D., Mladenovic D., Criscuoli M., Loria F., Veiman K.-L., Zocco D., Koort K., Zarovni N. (2021). Opportunities and Pitfalls of Fluorescent
Labeling Methodologies for Extracellular Vesicle Profiling on High-Resolution
Single-Particle Platforms. Int. J. Mol. Sci..

[93] Sancho-Albero M. (2023). Superfluorinated Extracellular Vesicles for In Vivo Imaging by (19)­F-MRI. ACS Appl. Mater. Interfaces.

[94] Ghodasara A., Raza A., Wolfram J., Salomon C., Popat A. (2023). Clinical Translation
of Extracellular Vesicles. Adv. Healthc. Mater..

[95] Dai S. (2008). Phase I clinical trial
of autologous ascites-derived exosomes combined with GM-CSF for colorectal
cancer. Mol. Ther..

[96] Sun L. (2016). Safety
evaluation of exosomes derived from human umbilical cord mesenchymal
stromal cell. Cytotherapy.

[97] Niu W. (2021). A Biomimetic Drug Delivery
System by Integrating Grapefruit Extracellular
Vesicles and Doxorubicin-Loaded Heparin-Based Nanoparticles for Glioma
Therapy. Nano Lett..

[98] Jia G. (2018). NRP-1 targeted and cargo-loaded
exosomes facilitate simultaneous
imaging and therapy of glioma in vitro and in vivo. Biomaterials.

[99] Yang Z. (2021). A New Nanomaterial Based
on Extracellular Vesicles Containing Chrysin-Induced
Cell Apoptosis Through Let-7a in Tongue Squamous Cell Carcinoma. Front. Bioeng. Biotechnol..

[100] Wu H. (2021). Extracellular-vesicles delivered tumor-specific
sequential
nanocatalysts can be used for MRI-informed nanocatalytic Therapy of
hepatocellular carcinoma. Theranostics.

